# Homozygous factor V leiden mutation: Rare etiology of pulmonary embolism

**DOI:** 10.1016/j.amsu.2022.104569

**Published:** 2022-09-13

**Authors:** Zakaria el marraki, Adam bouzhir, Zidane eddhima, Alaa-Eddine el bouanani, Najat mouine, Atif benyass

**Affiliations:** a Department of Clinical Cardiology, Morocco; b Department of Cardiac Intensive Care, Morocco; c Department of Rythmology, Morocco; d Department of Non-invasive Explorations, Morocco; e Department of Cardiology, Military Hospital of Instruction Mohammed V, Faculty of Medicine and Pharmacy, RABAT, Morocco

**Keywords:** Pulmonary embolism, Case report, Homozygous factor V Leiden mutation, Thoracic angioscan

## Abstract

**Introduction and importance:**

Venous thromboembolic disease (VTE), which includes pulmonary embolism (PE) and deep vein thrombosis (DVT), is a major public health problem with high morbidity and mortality. The main risk factors for VTE are surgery, active cancer, immobilization, trauma or fracture, pregnancy and estrogen therapy. Genetic risk factors are also present and are dominated by the factor V Leiden mutation, which is present in 20% of VTE and in 2–5% of the general population with an annual incidence of 0.1% (Margaglione and Grandone, 2011; Ridker et al., 1995) [4,5]. This mutation can be heterozygous or homozygous, which is rarer. In this context, we report the case of a 37-year-old patient with no medical or surgical history and no notable risk factors who was admitted to the emergency room for the management of acute dyspnea at rest in connection with a bilateral proximal pulmonary embolism originating from a homozygous factor V Leiden mutation.

Despite the efforts of the World Health organization, pulmonary embolism remains a major cause of morbidity and mortality in our days, and the etiological assessment is performed in a very few cases, which makes the management standardized and not specific. That is why it is important to make an etiological assessment in a systematic way especially in young subjects for an optimal management and to avoid recurrences.

**Case presentation:**

Here, we report a rare case of a 37-year-old patient, who was admitted for the management of resting dyspnea related to bilateral proximal pulmonary embolism, in whom the etiological work-up was in favor of a homozygous factor V Leiden mutation. This case shows diagnostic difficulties and management of this rare disease.

## Highlights

• Pulmonary embolism is a frequent and potentially serious pathology.
• The etiological assessment of pulmonary embolism is not carried out in a systematic way which makes the management not optimal with the possibility of repeated pulmonary embolism.
• Long-term anticoagulation is the mainstay of treatment for pulmonary embolism secondary to factor V Leiden mutation.

## 1 Introduction

Acute pulmonary embolism (PE) is a major cause of death worldwide, with more than 100,000 deaths in 2018 [1]. It is the third leading cause of cardiovascular death in hospitalized patients in Western countries, after acute myocardial infarction and stroke [2,3]. it should be noted that Numerous risk factors for venous thromboembolic disease have been described, including genetic risk factors which are dominated by the factor V Leiden mutation which is present in 20% of VTE and in 2–5% of the general population with an annual incidence of 0.1%/year [4,5].

Pulmonary embolism following homozygous factor V of Leiden mutation is very poorly described in the literature and its management is still controversial and there are no precise guidelines. In this paper we report the case of a young patient with no medical history admitted to the emergency department for the management of a pulmonary embolism related to a homozygous factor V Leiden mutation.

Our case report was written according to CARE guidelines [13].

## 2 Case presentation

A 37-year-old patient was admitted to the emergency room for acute resting dyspnea associated with palpitations. He was initially referred to a general practitioner 3 days before, who put the patient on symptomatic treatment without improvement of the symptoms and then referred to our cardiology center for further treatment. The interrogation found a patient with no cardiovascular risk factor and no medical or surgical history. In addition, there were no family history of thromboembolic disease. On examination, the patient was conscious, of normal weight and height with a BMI of 21kg/m*2. Tachycardic with a heart rate of 110 beats/min, hemodynamically stable with a heart pressure that was a 114/71mmhg. He was polypneic with a respiratory rate of 24 breaths/min and an O2 saturation of 92% on room air. On cardiovascular examination there was no sign of heart failure either right or left, the pleuropulmonary examination was normal. The complementary physical examination was with no particularity.

The differential diagnoses included all the etiologies of acute dyspnea, i.e., asthma decompensation, pneumothorax, tamponade, acute lung edema.

**Fig. 1:**
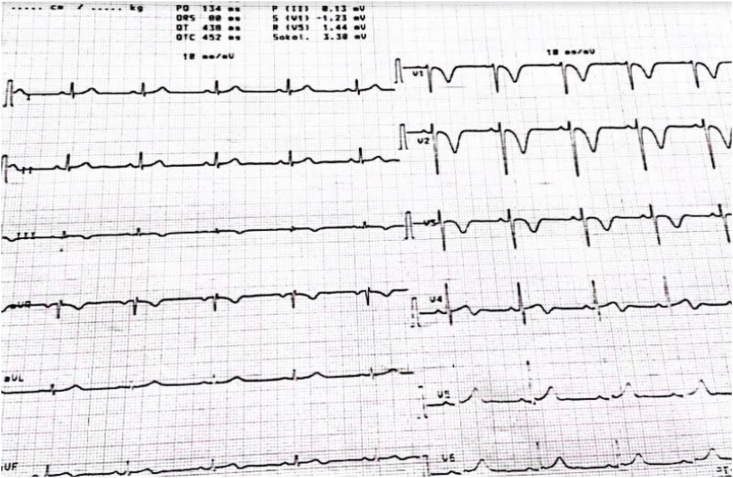
EKG showing negative T waves in the anteroseptal and inferior territories.

**Fig. 2:**
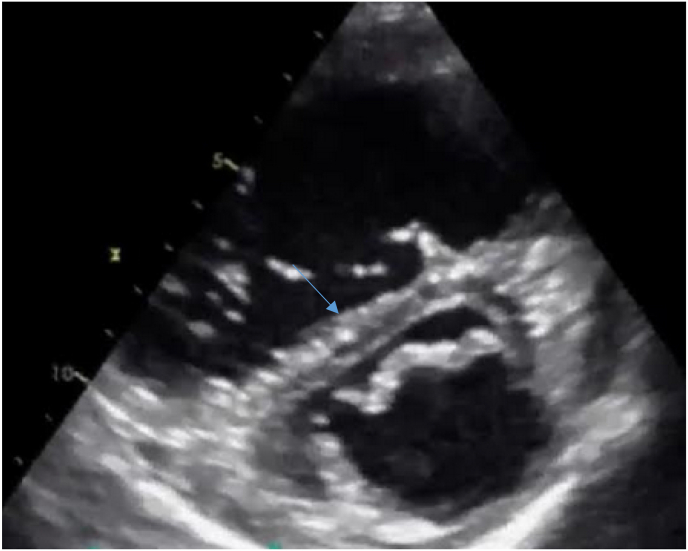
Short axis parasternal section showing dilatation of the right ventricle associated with flattening of the interventricular septum.

**Fig. 3:**
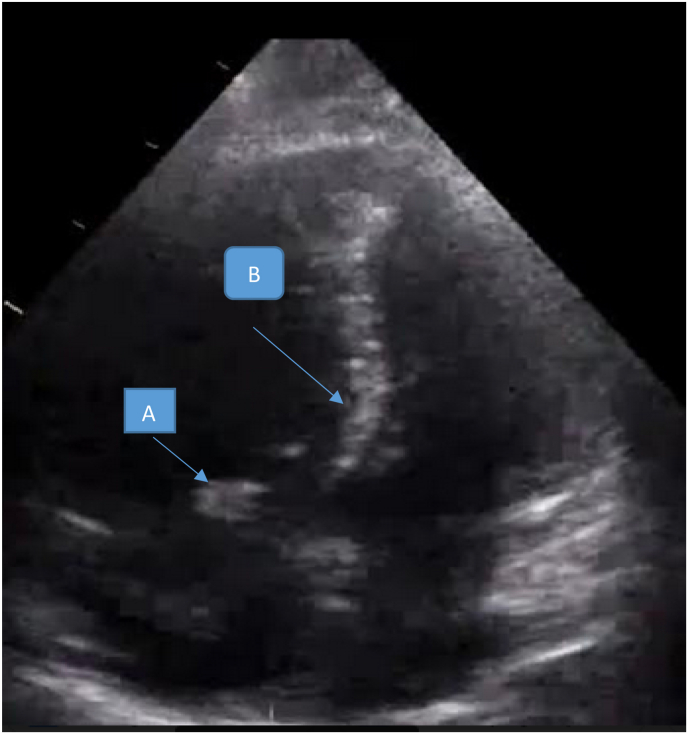
Apical section showing a right atrial thrombus(A), associated with a paradoxical septum(B).

**Fig. 4:**
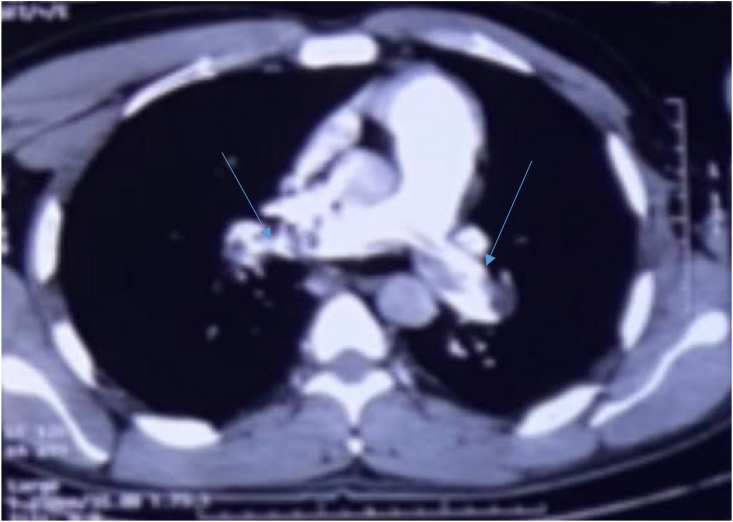
Thoracic angioscan showing bilateral proximal pulmonary embolism.

The EKG showed a regular sinus rhythm with a VR at 110 beats per minute, a constant PR at 116 ms and a fine QRS at 80 ms and negative T waves in anteroseptal and inferior (Fig. 1). The chest X-ray was unremarkable, the transthoracic echocardiography (TTE) objectified an aspect of an acute pulmonary heart with a non-dilated LV with good systolic function and a dilated Right ventricle (RV), the basal diameter of the RV was 50mm, RV/LV ratio was 1.3 non hypertrophied with paradoxical septum (Fig. 2) of altered longitudinal function TAPSE at 12mm, S wave at 7cm/s. there was also the presence of a right intra-atrial thrombus (see Fig. 3).

(Fig. 3). It was decided to perform a thoracic angioscan on the basis of the radiological findings and given the clinical context. The results showed the presence of a bilateral proximal pulmonary embolism (Fig. 4).

Biologically, his troponin on admission was borderline at 34pg/ml (normal<17pg/ml), but the D-dimer was at 5000ng/ml (normal<230ng/ml), the CRP was 25 mg/l (normal<5mg/l), hepatic assessment and renal function were slightly disturbed with a normalization during his hospitalization.

The patient was admitted to the cardiology intensive care unit where he was conditioned and scoped, the diagnosis of a pulmonary embolism was made on a bundle of arguments, echocariographic, scannographic, biological, electrical and clinical.

During his hospitalization, the patient underwent an etiological workup, given his young age and the severe nature of the pulmonary embolism, including an immunological workup and tumor markers that came back without any particularity, At the level of venous Doppler ultrasound of the lower limbs there was the presence of an aspect in favor of a DVT of the left popliteal vein extended to the superficial femoral vein, he had also benefited from a thrombophilia assessment which had objectified a resistance to activated protein C and a homozygous mutation of factor V of leiden.

The patient was hospitalized because of the high intermediate risk of The Simplified Pulmonary Embolism Severity Index (SPESI). He was treated with UFH at a dose of 3500 IU for a weight of 70kg in direct intravenous injection and then 35,000 IU/24 Hours for 5 days, he was subsequently put on oral anticoagulants + analgesic treatment.

It should be noted that the patient remained stable throughout his hospitalization and his dyspnea disappeared on the 6th day of his hospitalization, he stayed 7 days in intensive care of cardiology, then transferred to clinical cardiology, thereafter he was discharged and referred to the department of genetic medicine for possible exploration and was proposed a family screening for first degree relatives in search of a similar mutation and with a cardiological control in 1 month.

## 3 Discussion

The factor V leiden mutation is a major inherited risk factor for thrombophilia in the Western population, which is present in 20–25% of venous thromboembolic diseases. (4,5).it has been described that the risk of first venous thrombosis is 3–7 times higher in heterozygous carriers of the factor V Leiden mutation and 50 to 100 times higher in homozygous carriers compared with subjects without the mutation [[5], [6], [7]]. The association between pulmonary embolism and thrombophilia has been described in numerous studies, including a study performed at the Department of Vascular Surgery a (HGH), Qatar in this study 227 consecutive patients with a radiologically confirmed diagnosis of PE were included. Among them, 108 (47.6%) patients had a hypercoagulable state with at least one positive marker for thrombophilia [8]. Many authors have described that Pulmonary embolism occurs 10 years earlier in subjects with thrombophilia than in those without thrombophilia [8], and that The prevalence of symptomatic venous thromboembolic disease is higher in carriers of a homozygous factor V Leiden mutation 68% (n = 75) compared with 54% (n = 145) in carriers of a heterozygous factor V Leiden mutation [9].It should be noted that No study has been able to demonstrate the imputability between the factor V Leiden mutation and the severity of pulmonary embolism, but rather it is the association between several risk factors. Anticoagulation is the basic treatment for venous thromboembolic disease. Many patients with hereditary thrombophilia have received anticoagulation with either a vitamin K antagonist (VKA) or heparin [10]. The role of direct oral anticoagulants (DACs) in the treatment of hereditary thrombophilia remains unknown [11]. Cook et al. showed that a patient with ovarian vein thrombosis associated with an FVL mutation was successfully treated. In addition, a subgroup analysis by Schulman et al. demonstrated that dabigatran was non-inferior to warfarin in patients with hereditary thrombophilia with respect to venous thromboembolic disease (VTE) recurrence or VTE-related deaths [12]. In our case the patient was 37 years old and without risk factors or notable ATCD, he was initially admitted for the management of rest dyspnea related to a bilateral proximal pulmonary embolism, he was hospitalized in the cardiology intensive where he had benefited from a global etiological workup in favor of a homozygous mutation of factor V Leiden which unfortunately remains until now a poorly understood entity by practitioners and very few studies have been carried out in this sense, we decided to write this paper to share this rare case and the way in which it was managed, that was inspired from the different papers published in this sense and to encourage practitioners to do more studies in this direction to improve the prognosis and for an optimal management in this group of patients. our patient was put on rivaroxaban 15mg*2/d with a control appointment in one month.

## 4 Conclusion

- Pulmonary embolism is a frequent and sometimes serious pathology.
- The search for thrombophilia is systematic in young subjects.
- The management of pulmonary embolism is multidisciplinary.
- interest in information, education, and communication.

### 4.1 Learning objectives

1) to get an idea of the clinical presentation and the electrical and imaging aspects of this rare entity.
2) to have the reflexion to search for the genetic etiologies of pulmonary embolism especially in the young subject and also to propose a genetic test in the relatives of the first degree.

## Ethical approval

The ethical committee approval was not required give the article type (case report). However, the written consent to publish the clinical data of the patients was given and is available to check by the handling editor if needed.

## Sources of funding for your research

None.

## Author contribution

Zakaria El marraki: Study concept, Data collection, Data analysis, Writing the paper. Adam Bouzhir: Data collection, Data analysis. Zidane Eddehima: Data collection. Alaa-eddine el bouanani: Data collection. Najat Mouine: Supervision and data validation. Atif Benyass: Supervision and data validation.

## Trail registry number

This is not an original research project involving human participants in an interventional or an observational study but a case report. This registration is was not required.

## Guarantor

Zakaria El marraki.

## Provenance and peer review

Not commissioned, externally peer-reviewed.

## Consent

Written informed consent was obtained from the patient for publication of this case report. CARE guidelines were applied for reporting this case report’ finding.

## Declaration of competing interest

The authors declare no conflict of interest.
